# Genome-wide association study for empirically derived metabolic phenotypes in the Framingham Heart Study offspring cohort

**DOI:** 10.1186/1753-6561-3-s7-s53

**Published:** 2009-12-15

**Authors:** Marsha Wilcox, Qingqin Li, Yu Sun, Paul Stang, Jesse Berlin, Dai Wang

**Affiliations:** 1 Epidemiology, Johnson & Johnson Pharmaceutical Research and Development, LLC, 1125 Trenton-Harbourton Road, PO Box 200, M/S K304, Titusville, New Jersey 08560 USA; 2 Pharmacogenomics, Johnson & Johnson Pharmaceutical Research and Development, LLC, Raritan, New Jersey 08869 USA

## Abstract

We used data reduction and clustering methods to identify five phenotypically homogeneous groups of study participants with similar profiles for cardiovascular disease risk factors. We constructed both qualitative (binary subgroup membership) and quantitative traits (probability of subgroup membership) for each individual. The Cluster 1 comprised individuals who were generally healthy and had some history of smoking. Cluster 2 was dropped from the analyses due to the preponderance of missing data. Cluster 3 was used as the control group, healthy non-smokers. Members of Cluster 4 had features of the metabolic syndrome and were generally not as obese as Cluster 5. Obesity was the hallmark of Cluster 5, the members of which also had some features of the metabolic syndrome.

We then examined the genetic associations with both qualitative and quantitative representations of these empirically derived traits. Genetic analyses of the qualitative traits were conducted, comparing each of the affected groups with the unaffected cluster alone and, to increase statistical power, the unaffected group and healthy smokers combined. One single-nucleotide polymorphism on chromosome 4 met a conservative genome-wide significance level, but the effect was muted when we accounted for population stratification. The results for the quantitative traits were similar, with a small number of genome-wide significant findings muted by control for admixture. The directional findings will provide the basis for hypothesis generation for syndromes such as the metabolic syndrome and obesity.

## Background

The identification of subtypes of disease using data reduction and clustering methods has been helpful for identifying genetic associations in schizophrenia, rheumatoid arthritis, and other disorders [1-3].

The Framingham Heart Study (FHS) is an ongoing longitudinal study focusing on the development of coronary heart disease and associated risk factors in Framingham, MA. The long history of research conducted in Framingham has contributed to the contemporary understanding of cardiovascular and related diseases. One advantage of this study is that the ascertainment criteria do not require disease at the time of study entry. In addition, this is a random sample of households and not restricted to those seeking healthcare for any specific complaint.

Our analyses of the data made available to participants in the Genetic Analysis Workshop (GAW) 16 were restricted to members of the Offspring Cohort because some measures of metabolic function were not available in the Original Cohort and were not available for more than one measurement period in the Generation 3 Cohort.

The objectives of this study were 1) to identify subgroups of study participants with similar phenotypic characteristics (or syndromes) including patterns across measurements at multiple visits, and 2) to construct qualitative and quantitative representations of the new phenotypic subgroups, and 3) to conduct genome-wide association analyses of the quantitative traits; and for qualitative traits, to compare the newly identified affected phenotypic groups with unaffected controls.

## Methods

### Phenotypic data

We created categorical variables using the Centers for Disease Control (CDC) definitions for each of the following for each exam: body mass index (BMI; underweight, normal weight, overweight, or obese), high cholesterol (measured or on lipid lowering medication), low high-density lipoprotein (HDL), high triglycerides, and hypertension (measured or on medication) [4]. These variables, along with categorical representations of smoking (current, ever, never), diabetes (yes, no), treatment for hypertension, treatment for high cholesterol, and heart disease (yes, no), were the basis for the data reduction and clustering.

### Phenotype definitions

The strategy for the development of qualitative traits included nonparametric data reduction, iterative two-staged clustering on the observed dimensions, and the assignment of binary membership in each cluster for each individual. Quantitative traits (probability of cluster membership) were estimated using logistic regression with cluster membership as the outcome and all variables used for clustering as predictor variables in the models.

We used multiple-correspondence analysis (MCA) for data reduction instead of the more traditional principal-components analysis (PCA). PCA is a method commonly used for data reduction. These data did not meet the distributional assumptions for a Pearson correlation, the basis for PCA. A similar method designed for use with categorical data was employed. MCA is a nonparametric data reduction method free of the assumptions underlying PCA and was developed for qualitative data. The objective of MCA is to identify a low-dimensional subspace that comes closest to all of the data points. It is analogous to graphing the results of a factor analysis in a multidimensional euclidean space. However, the space identified in MCA is not euclidian. The coordinates of each individual in the identified multi-dimensional space served as the basis for the identification of subgroups or clusters [5].

Each study participant with phenotype data on two or more visits was assigned a score on each of the 22 dimensions retained based upon the eigenvalues (data not shown). Next, a multi-staged clustering strategy was used to identify distinct subgroups [6]. It is not unusual for groups identified with clustering techniques to be subject to the idiosyncrasies of the estimation data set. In an attempt to mitigate that difficulty, we first conducted repeated *k*-means clustering with different random cluster seeds and used a larger *k *(number of clusters) than we expected in the data. Groups that consistently clustered together across all of the initial analyses were identified as intact clusters. An agglomerative hierarchical clustering algorithm was then implemented using the intact clusters and the remaining individuals in the sample. An examination of the change in Ward's aggregation criterion and the nature of the groups was used to choose the final cluster structure [5,6]. SPAD software [7] was used for both the MCA and the clustering algorithms. SAS software [8] was used to compute quantitative traits and for subgroup comparisons.

### Genotype data preparation

There were 6,848 individuals with genotype data from Affymetrix 500 k platform (500,568 single-nucleotide polymophisms (SNPs)). Quality control for the genotype data was conducted at both subject and SNP level. At the subject level, we retained subjects with call rates greater than 0.90. Sex discrepancies were evaluated using the heterozygosity rate of X-chromosome SNPs and comparing with the phenotype data. Only subjects from the second generation were kept, and one subject from each pair of family members or cryptic relatedness was retained in the subsequent analysis. At the SNP level, we retained SNPs with a call rate greater than 0.90, a minor allele frequency of at least 0.01, and Hardy-Weinberg equilibrium *p*-value greater than 9.99 × 10^-8 ^(i.e., 0.05/(no. of SNPs tested)). We also excluded SNPs that could not be mapped to reference genome assembly. After data quality control, there were 1,754 subjects and 418,411 SNPs retained in the final dataset.

### PCA

PCA was used to examine population stratification. This analysis was performed using EIGENSOFT 2.0 [9,10]. Theoretically, the leading principal component should reflect population structure. We noticed that some of the leading axes appeared to be dominated by a set of markers in a very small region that showed extended linkage disequilibrium. To deal with this problem, we applied a modified version of the PCA as described by Fellay et al. [11]. The method we used is described in detail by Wang et al. [12].

PCA was performed in each of the analysis sets separately to derive significant principal components that represented the population structure in the analysis set. Population stratification (admixture) was controlled in subsequent analyses by including the final set of 18 significant components as covariates.

### Genome-wide association (GWA) analyses

GWA analyses were performed using PLINK 1.03 [13]. An additive genetic model was assumed for all GWA analyses. Each of the newly derived affected subgroups was compared with the identified control group separately using allelic chi-square tests in the absence of principal-component adjustment and logistic regression, including significant principal components derived above as covariates to control for admixture. A threshold of 4.2 × 10^-7 ^was used for genome-wide significance based on a Bayesian formula as described by Lencz et al. [14].

## Results

### Phenotypes

Among the 2,760 study participants in the Offspring Cohort, five clusters were identified. Cluster 1 comprised individuals who were generally healthy and had some history (current or past) of smoking (*n *= 949, 34.4% [of the sample]). Cluster 2 (*n *= 365, 13.2%) comprised individuals who were missing information on two or more measurements. This group was omitted from the genetic analyses. Cluster 3 comprised healthy non-smokers (*n *= 597, 21.6%). This group served as the control group in our genetic association studies. Cluster 4 (*n *= 376, 13.6%) comprised individuals with features of the metabolic syndrome (MS) who were generally not obese (using the Centers for Disease Control definition of BMI of 30 or greater [14]). Obesity was the hallmark of the Cluster 5 (*n *= 473, 17.1%), the members of which also had some features of the MS. MS, as identified by the National Cholesterol Education Program's Adult Treatment Panel III, is a clustering of risk factors that can lead to cardiovascular disease (CVD). The risk factors include: abdominal obesity, atherogenic dyslipidemia, raised blood pressure, insulin resistance with or without glucose intolerance, proinflammatory state, and prothrombotic state [15].

**Figure 1:**
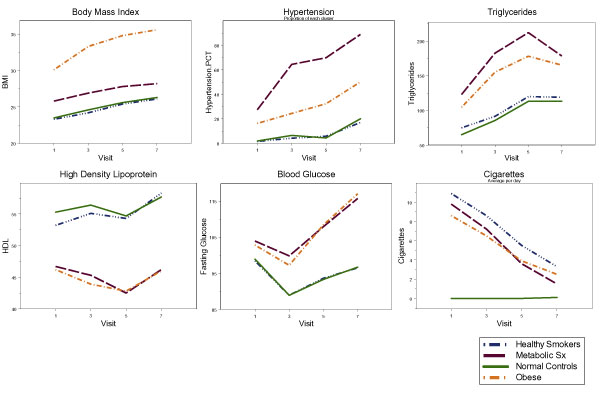
**Cluster-specific phenotypic characteristics across visits**.

The distributions of sex, age, diabetes ever and myocardial infarction ever are as follows (% female, average age at visit 1, % diabetes, % myocardial infarction): healthy smokers (60.0%, 32.8, 2.6%, 2.2%); healthy controls (59.5%, 32.9, 2.2%, 1.7%); MS (61.1%, 39.5, 28.2%, 25.2%); obese (42.7%, 39.7, 24.5%, 7.4%). By Visit 7, nearly 90% of the MS group was on treatment for hypertension. The same was true for 45% of the Obese group, as well as 12% of the healthy smokers and 18% of the controls. Figure 1 shows descriptive statistics for the phenotypic groups across visits available for this study.

### Genome-wide association

We compared the MS and Obese groups with the healthy control group, and, to increase power (power calculations not shown), with the healthy control and healthy smokers combined. We used a fairly conservative approach to correct population stratification (18 principal components). Neither of the analyses using the controls alone revealed SNPs with *p*-values at or beyond the genome-wide significance level (data not shown). There was one genome-wide significant SNP on chromosome 4 when we used the larger comparison group. However, this finding was muted when we accounted for population stratification.

**Figure 2:**
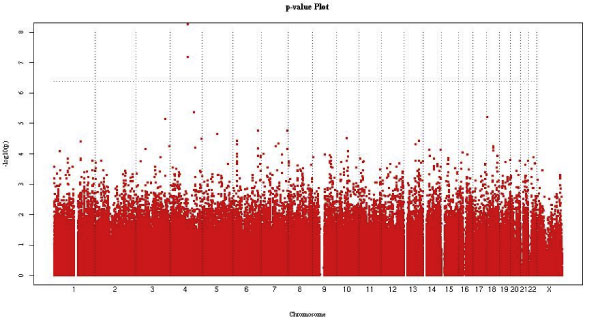
**GWAS: quantitative traits for metabolic syndrome and obese groups**.

Figure 2 shows the GWAS results for the quantitative traits for the two affected groups. As was the case with the qualitative traits, there were significant findings in a region on chromosome 4 that were muted when the analyses accounted for population structure using principal components estimated for that purpose.

Our analyses of the 50 k chip showed similar results (data not shown). For the quantitative trait for the MS-like group there were five results across the genome with *p *< 1 × 10^-5^; similarly, there were eight for the Obese group. In both cases the findings were somewhat muted when we accounted for admixture.

## Discussion

We identified five phenotypic clusters using a limited set of measures pertaining to medical history, metabolic function, and environmental exposures. One group was omitted due to missing data. Two groups appeared to be relatively healthy, one of which was more inclined toward tobacco use than the other. The remaining two groups were characterized by elevated measures related to MS and obesity. Interestingly, those characterized by features of MS were not as heavy, nor did they gain weight as quickly over time as did the Obese group. Features associated with MS were not as prevalent in the group characterized by obesity as they were in the other affected cluster.

There are several limiting factors in our analyses. We used a conservative approach for the correction of population stratification. We also used a somewhat conservative approach for genome-wide significance levels. It would be interesting to see these results using an empirical *p*-value instead.

Next steps in these analyses will be to use the results for hypothesis generation and to also examine regions with suggestive findings for genes that have been implicated in metabolic disorders and obesity.

## Conclusion

We identified distinct clusters of individuals with different manifestations of metabolic disorders. Genetic association analyses revealed several regions for further investigation.

## List of abbreviations used

BMI: Body mass index; CVD: Cardiovascular disease; FHS: Framingham Heart Study; GAW: Genetic Analysis Workshop; GWA: Genome-wide association; HDL: High density lipoproteins; MCA: Multiple correspondence analysis; MS: Metabolic syndrome; PCA: Principal-components analysis; SNPs: Single-nucleotide polymorphisms

## Competing interests

The authors declare that they have no competing interests.

## Authors' contributions

MW designed the study, conducted the phenotype analyses, and drafted the manuscript; QL carried out all of the genetic analyses and GWAS figure preparation; YS participated in discussions about the analytic methods; PS and JB participated in discussions about the phenotype definitions and facilitated acquisition of the data; DW participated in discussions about the analytic methods for the GWAS.
